# The impact of biological sex and stroke volume constituents on heterogeneity in the $\dot{Q}$–${\dot{V}}_{O_{2}}$ relationship and skeletal muscle saturation during cycling

**DOI:** 10.1113/EP093845

**Published:** 2026-07-22

**Authors:** Adam N. Di Salvo, Sinan Osman, Jacob L. Schwartz, Nino Nikolovski, Robert F. Bentley

**Affiliations:** ^1^ Faculty of Kinesiology and Physical Education University of Toronto Toronto Ontario Canada

**Keywords:** echocardiography, exercise, interindividual variability, left ventricle, oxygen delivery

## Abstract

Interindividual variability in the cardiac output ($\dot{Q}$) to the rate of oxygen consumption (${\dot{V}}_{O_{2}}$) slope ($\dot{Q}$–${\dot{V}}_{O_{2}}$) in healthy males is related to stroke volume (SV); however, the impact of differences in left ventricular (LV) structure and function as well as biological sex on this slope is unclear. The $\dot{Q}$–${\dot{V}}_{O_{2}}$ slope, LV function, convective oxygen delivery and vastus lateralis skeletal muscle oxygen saturation were assessed in 42 untrained, healthy individuals (25 ± 5 years, 50% female) during submaximal semi‐upright cycling up to 120 W, followed by progressive exercise to exhaustion. There was considerable variability in the submaximal $\dot{Q}$–${\dot{V}}_{O_{2}}$ slope (∆40–∆80 W; 1.8–11.8 L of blood/min per L of O_2_/min), though males and females did not differ (6.0 ± 2.2 vs. 4.9 ± 2.2 L of blood/min per L of O_2_/min; *P* = 0.140) despite males having a greater calculated blood volume (*P *< 0.0001). The $\dot{Q}$–${\dot{V}}_{O_{2}}$ slope was correlated positively to skeletal muscle desaturation (all *r* > 0.550, all *P *< 0.029) and negatively to arterial oxygen content (*r* = −0.619, *P* = 0.005) in females only. Accounting for differences in filling time, SV heterogeneity was explained by preload, contractility, and biological sex (*F* = 36.11; *P *< 0.0001, *r^2^* = 0.800). These findings suggest that the $\dot{Q}$–${\dot{V}}_{O_{2}}$ slope in females is influenced by arterial oxygen content and impacts skeletal muscle oxygenation. This highlights potential biological sex differences in the underlying mechanisms matching oxygen delivery to oxygen demand, with females appearing more dependent upon skeletal muscle oxygen extraction to compensate for lower arterial oxygen content and blood volume. Further, SV heterogeneity in the $\dot{Q}$–${\dot{V}}_{O_{2}}$ slope is explained by preload and contractility, but not afterload.

## Abstract (highlights)

- **What is the central question of this study?**
  What is the effect of biological sex on the relationship between arterial oxygen content and skeletal muscle oxygenation to $\dot{Q}$–${\dot{V}}_{O_{2}}$ heterogeneity and what is the contribution of left ventricular structure/function to heterogeneity in stroke volume?
- **What is the main finding and its importance?**
$\dot{Q}$–${\dot{V}}_{O_{2}}$ slope was unaffected by biological sex, but correlated with arterial oxygen content and skeletal muscle oxygenation in females. Heterogeneity in stroke volume was predicted by surrogates of preload and contractility alongside biological sex. This suggests distinct mechanisms underscoring cardiovascular responses facilitating the matching oxygen delivery to oxygen demand between sexes.

## 1 INTRODUCTION

Matching oxygen delivery to the active skeletal muscle's oxygen demand during continuous aerobic exercise is critical as inadequate oxygen delivery impairs performance (Amann et al., 2007) and increases ratings of perceived exertion (RPE) (MacNutt et al., 2015). Increases in convective oxygen delivery, the product of cardiac output ($\dot{Q}$) and arterial oxygen content ($C_{aO_{2}}$) (McLellan & Walsh, 2004), arise from $\dot{Q}$ (Andersen & Saltin, 1985; Proctor et al., 1998) as $C_{aO_{2}}$ remains stable throughout exercise in healthy individuals (Andersen & Saltin, 1985). During exercise, the rate of oxygen consumption (${\dot{V}}_{O_{2}}$) increases as more energy is required to perform work (Astrand et al., 1964) and as such, $\dot{Q}$ and ${\dot{V}}_{O_{2}}$ are intimately related. Their interaction can be quantified as the $\dot{Q}$–${\dot{V}}_{O_{2}}$ relationship, which represents how much $\dot{Q}$ must increase to facilitate an increase in ${\dot{V}}_{O_{2}}$ by 1 L/min. Convention states that $\dot{Q}$ increases by ∼5–7 L/min per litre of ${\dot{V}}_{O_{2}}$ (Adami et al., 2014; Faulkner et al., 1977; Proctor et al., 1998) with interindividual differences in the $\dot{Q}$–${\dot{V}}_{O_{2}}$ slope attributed to differences in $C_{aO_{2}}$ (Adami et al., 2014). However, conflicting reports have found variation in $\dot{Q}$ up to ∼5 L/min at any given rate of submaximal ${\dot{V}}_{O_{2}}$ (Astrand et al., 1964; Reeves et al., 1961; Yamaguchi et al., 1986) with recent work by Bentley et al. (2018) identifying variability in $\dot{Q}$ at a given submaximal ${\dot{V}}_{O_{2}}$ to be completely independent of $C_{aO_{2}}$ in healthy males. Interestingly, phenotypically higher $\dot{Q}$ responses were mediated by elevations in stroke volume (SV) and despite differences in $\dot{Q}$, active muscle perfusion was seemingly independent as local active skeletal muscle oxygen saturation (Δ$S_{mO_{2}}$) was unaffected.

SV represents an interdependent relationship between preload, contractility, afterload and heart rate (HR) (Silverthorn, 2019). While contractility and preload both increase during aerobic exercise (Braunwald, 1965; Ross & Braunwald, 1964) and facilitate enhancements in left ventricular (LV) function, elevations in HR reduce filling time (Higginbotham et al., 1986) while increases in afterload elevate the resistance to open the aortic valve and challenge LV function (Imperial et al., 1961). Additionally, how the factors impacting LV function may contribute to a phenotypically greater SV in some individuals compared to others at the same submaximal ${\dot{V}}_{O_{2}}$ remains to be comprehensively explored.

While previous research by Proctor et al. (1998) in chronically endurance trained individuals identified the $\dot{Q}$–${\dot{V}}_{O_{2}}$ slope to be independent of biological sex, females presented with an attenuated ability to maintain SV at higher submaximal intensities while males did not. Differences in LV structure and function have been documented between males and females (Fu et al., 2004; Pianosi et al., 2018; Plotnick et al., 1986); however, their effect on the $\dot{Q}$–${\dot{V}}_{O_{2}}$ slope is unknown. In addition to differences in cardiac structure and function, while females typically present with a lower haemoglobin concentration ([Hb]), reduced oxygen‐carrying capacity (Astrand et al., 1964; Proctor et al., 1998) and greater vastus lateralis skeletal muscle desaturation during incremental cycling (Espinosa‐Ramírez et al., 2021) compared to males, how this relates to the $\dot{Q}$–${\dot{V}}_{O_{2}}$ slope remains to be elucidated.

Therefore, the purpose of this study was first to explore the effect of biological sex on the relationship between $C_{aO_{2}}$ and Δ$S_{mO_{2}}$ to $\dot{Q}$–${\dot{V}}_{O_{2}}$ heterogeneity in untrained healthy individuals, and second, to examine the contribution of LV structure and function to heterogeneity in SV and the $\dot{Q}$–${\dot{V}}_{O_{2}}$ slope. It was hypothesized that females will have a greater $\dot{Q}$–${\dot{V}}_{O_{2}}$ slope to account for lower [Hb] and $C_{aO_{2}}$, while Δ$S_{mO_{2}}$ will be independent of the $\dot{Q}$–${\dot{V}}_{O_{2}}$ slope in both sexes. It was further hypothesized that greater LV preload, contractility, and a reduced afterload will contribute to a greater SV and submaximal $\dot{Q}$–${\dot{V}}_{O_{2}}$ slope.

## 2 METHODS

### 2.1 Ethical approval

The University of Toronto Health Sciences research ethics board approved this study (no. 43107) according to the terms of the *Declaration of Helsinki* and all participants provided written informed consent before their participation.

### 2.2 Participants and sample size estimation

Forty‐two healthy, recreationally active (<3 h/week structured exercise) males and females participated in this study (25 ± 5 years, 50% female). Participants were included if they were: (1) between the ages of 18 and 35, (2) had a body mass index <29.9 kg/m^2^, (3) self‐reported as healthy with successful completion of a Physical Activity Readiness Questionnaire for Everyone (PAR‐Q+) (Jamnik et al., 2011), and (4) had no past history of diagnosis of COVID‐19 that required hospitalization. Participants were excluded if they were: (1) unable to perform cycling exercise, (2) regularly used tobacco, (3) had any prior history of cardiovascular or pulmonary disease, or (4) were unable to provide written informed consent. Expecting a similar level of variability in SV and the slope of the $\dot{Q}$–${\dot{V}}_{O_{2}}$ relationship as in previous work by Bentley et al. (2018), 21 male and 21 female participants are required to explore SV and $\dot{Q}$–${\dot{V}}_{O_{2}}$ variability. Additionally, SV has been shown to be correlated with surrogate measures of preload (end diastolic volume, EDV; *r* = 0.93; Nixon et al., 1982), contractility (global longitudinal strain, GLS; *r* = 0.75; El‐Dosouky et al., 2023), and afterload (systolic blood pressure, SBP; β = 0.2; Nayor et al., 2023). Expecting a moderate correlation (*r* = 0.45) (Cohen, 1992) between factors impacting LV function, with a power of 0.8 and an α of 0.05, 36 recreationally active (<3 h/week structured exercise) young adults are required to explore heterogeneity in SV due to factors impacting LV function and the $\dot{Q}$–${\dot{V}}_{O_{2}}$ slope.

### 2.3 Experimental design

Participants reported to the laboratory for a single data collection session consisting of incremental cycling exercise with concomitant transthoracic echocardiography. Participants were asked to refrain from exercise for 24 h, caffeine and/or any energy‐altering substances for 12 h, and food for 4 h prior to their laboratory visit. Hydration status was not collected, though participants were able to drink water ad libitum prior to arrival. Upon arrival at the laboratory, standard anthropometric measurements were obtained from each participant. Age, height and weight were obtained as well as skinfold thickness of the right vastus lateralis ∼14 cm above the patella. Venipuncture at the antecubital vein (1 mL) was performed to assess [Hb] and compute oxygen delivery. A 7‐day physical activity recall questionnaire adapted from Sarkin et al. (1997) was completed to confirm current physical activity levels and compute weekly metabolic equivalent of task (METs).

### 2.4 Cycling exercise

**FIGURE 1:**
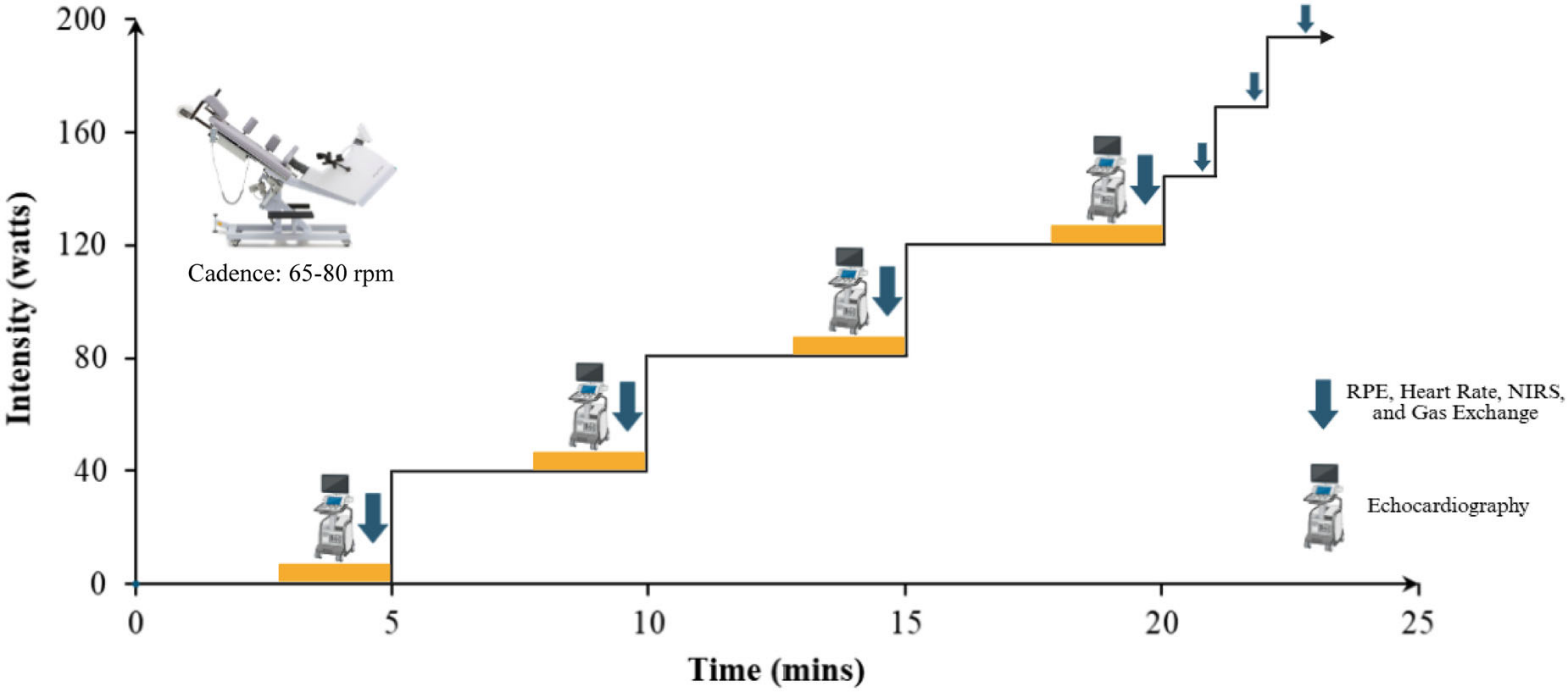
Visual schematic of exercise protocol. Following 5 min of rest, submaximal exercise was completed at 40, 80 and 120 W until minute 20. Afterwards, intensity increased by 25 W/min until volitional exhaustion. All exercise was completed in a semi‐upright body position (∼40° incline, ergometer pictured). Echocardiography was recorded at rest and during the last 3 min of each exercise stage up to 120 W (orange rectangle). Continuous measures collected included whole body ${\dot{V}}_{O_{2}}$, near‐infrared spectroscopy (NIRS) and heart rate. Data were analysed as an average of the last 30 s of each stage (blue arrow). Intermittent measures were recorded at rest and each exercise intensity (blue arrow, last 30 s) and include ratings of perceived exertion (RPE).

Participants completed three 5‐min stages of submaximal cycling at 40, 80 and 120 W in a semi‐upright body position on a tilt‐recline stress echocardiography table ergometer (40° incline with no tilt; Ergoline, Ergoselect 1200E, Bitz, Germany) (Figure 1). Following this, the intensity was then increased by 25 W every minute until volitional exhaustion. Participants self‐selected a cadence between 65 and 80 rpm and maintained this throughout exercise. Exhaustion was defined as failure to maintain their selected cadence within 5 rpm for >5 s. Seat position was adjusted for each participant to ensure individual comfort and a consistent cycling position (slight bend in the knee at full pedal extension). Prior to exercise, 5 min of semi‐upright rest was completed.

### 2.5 Transthoracic echocardiography

Two‐dimensional, transthoracic echocardiography (GE Vivid E9 Imaging System, GE Medical, Horten, Norway) was performed by an experienced, trained research sonographer using a M4S Matrix Sector Array probe (2–5 MHz). Images were acquired at semi‐upright rest and during the final 3 min of each exercise intensity up to 120 W. Apical two, four and five‐chamber along with parasternal long and short axis images were acquired at 60–90 fps to assess LV morphology and LV volumes. Cardiac dimensions, volumes, diastolic function and speckle‐tracking derived measures of LV strain were completed up to intensities eliciting HRs prior to when sinus tachycardia results in the fusion of early (*E*) and late (*A*) mitral inflow velocities (Chung et al., 2004).

LV EDV and end systolic volume (ESV) were measured in the apical two‐chamber and apical four‐chamber views using the modified bi‐plane Simpson's method (Lang et al., 2015). EDV could not be determined in two individuals at the 80 W intensity due to lateral wall dropout. SV was quantified with LV outflow tract (LVOT) diameter and the velocity time integral (VTI) because it better approximates gold standard cardiac magnetic resonance imaging measures of SV compared to 2D volumetric measurement techniques (Cowie et al., 2023). SV could not be computed for one individual at both rest and 40 W due to a missing LVOT VTI image. Longitudinal strain, which has a strong correlation with SV, $\dot{Q}$ and subclinical reductions in LV pump function (El‐Dosouky et al., 2023), was assessed using speckle‐tracking echocardiography, in which endocardial borders were traced using a point‐and‐click method on apical four chamber. Border tracking was previewed prior to processing to allow for visual confirmation of true endocardial tracking. A standard three‐lead electrocardiogram was recorded to allow cardiac gating during echocardiographic analysis. All images were analysed offline by a single observer and averaged over three cardiac cycles using dedicated software (EchoPAC™ v113, GE Medical, Horten, Norway) in accordance with the American Society of Echocardiography (Lang et al., 2015). The observer completing analysis had an inter‐day coefficient of variation for EDV, GLS and LVOT VTI (*n* = 10) of 4.4%, 8.5% and 1.6%, respectively.

### 2.6 Skeletal muscle oxygen saturation

Skeletal muscle oxygen saturation of the right vastus lateralis muscle was measured by continuous wave near‐infrared spectroscopy (Moxy NIRS; Fortiori Design LLC, Hutchinson, MN, USA) at a sampling frequency of 2 Hz. This measure represents the balance between blood flow into the capillaries and extraction of oxygen by muscle through diffusion. This device has a coefficient of variation during 100 W of cycling of 4% (Crum et al., 2017) and employs four wavelengths of near‐infrared light (680, 720, 760 and 800 nm). Based upon light detector and emitter distances, this device has a penetration depth of 12.5 mm and provides relative changes in the proportional concentrations of oxygenated and deoxygenated haemoglobin/myoglobin ($S_{mO_{2}}$ = oxy‐[Hb/Mb]/total [Hb/Mb], %). The device was secured to the skin over the vastus lateralis muscle belly ∼14 cm above the superior border of the patella with adhesive tape and covered with a light blocking elastic bandage. Manual palpation of the vastus lateralis muscle belly guided individual placement adjustments due to leg anthropometrics, and final positioning with correct device placement was confirmed during a brief isometric quadriceps contraction through the presence of desaturation. Δ$S_{mO_{2}}$ was measured continuously from rest to exercise cessation. Δ$S_{mO_{2}}$ in seven females could not be analysed due to excess skinfold thickness (>12.5 mm) at the vastus lateralis.

### 2.7 ${\dot{V}}_{O_{2}}$, arterial saturation, arterial blood pressure and RPE

Whole body ${\dot{V}}_{O_{2}}$ was continuously measured breath‐by‐breath using a calibrated, computer‐based system (Vmax Encore 229, CareFusion, Yorba Linda, CA, USA) as described previously (Bentley et al., 2025). Participants were fitted with 7450 V2 series reusable Hans Rudolph (Shawnee, KS, USA) mask with a Medgraphics (Tewkesbury, Gloucestershire, England) mask adapter. Arterial pulse saturation was measured continuously by pulse oximetry (ChoiceMMed; Beijing Choice Electronic Technology Co., Ltd, Beijing, China) on the index finger of the right hand. Arterial blood pressure was measured at the left arm using an automated auscultatory device (Tango M2, SunTech Medical, Morrisville, NC, USA) at rest and each exercise intensity. This device has been specifically designed for use during exercise testing and is not susceptible to exercise‐induced movement artifact (Cameron et al., 2004). Participants were also asked to rate their subjective physical exertion by pointing at the Borg RPE 6–20 scale (Borg, 1970). Prior to exercise participants were oriented to the scale and read a standardized script modified from Faulkner & Eston (2007) that instructed participants to rate their total amount of fatigue, combining all sensations and feelings of physical stress, effort and fatigue, without focusing on any one factor, such as leg pain, shortness of breath or exercise intensity.

### 2.8 Data analysis

#### 2.8.1 ${\dot{V}}_{O_{2}}$, skeletal muscle saturation, blood pressure and RPE

At rest and each exercise intensity, the final 30 s was averaged for ${\dot{V}}_{O_{2}}$ and Δ$S_{mO_{2}}$ to ensure participants were in steady state, and that data were collected after the completion of ultrasound image acquisition. ${\dot{V}}_{O_{2}}$ peak was quantified as the highest 30 s average ${\dot{V}}_{O_{2}}$ achieved. Arterial blood pressure and RPE were also assessed during the final 30 s of each stage.

#### 2.8.2 Arterial–venous oxygen difference and oxygen cost

The arterial–venous oxygen difference (a‐vO_2_) was computed by dividing ${\dot{V}}_{O_{2}}$ by $\dot{Q}$. Oxygen cost was calculated as the change in ${\dot{V}}_{O_{2}}$ from rest divided by cycling stage in watts.

#### 2.8.3 Submaximal $\dot{Q}$–${\dot{V}}_{O_{2}}$ identification

A linear regression was completed for each participant's Δ$\dot{Q}$ versus Δ${\dot{V}}_{O_{2}}$ from 40 to 80 W to identify an individual's $\dot{Q}$–${\dot{V}}_{O_{2}}$ slope. This two‐intensity approach aligns with previous work (Proctor et al., 1998) and was selected because the 120 W stages were determined to not be submaximal in nature (relative intensity was ≥90% of ${\dot{V}}_{O_{2}}$ peak in 16 participants). One participant could not be analysed due to a missing LVOT VTI measurement at rest.

#### 2.8.4 Identification of relative heterogeneity in $\dot{Q}$–${\dot{V}}_{O_{2}}$

Relative heterogeneity in $\dot{Q}$–${\dot{V}}_{O_{2}}$ was calculated to express percentage variability around the mean: (individual $\dot{Q}$–${\dot{V}}_{O_{2}}$ slope/group average) × 100.

#### 2.8.5 Venous blood sampling and computation of arterial oxygen content and oxygen delivery, and blood volume

Haemoglobin concentration was assessed with oximetry (Avoximeter 1000e, Instrumentation Laboratory USA; Werfen, Bedford, MA, USA). Arterial oxygen content ($C_{aO_{2}}$) was computed as $C_{aO_{2}}$ = (Hb × 1.34) × ($S_{aO_{2}}$/100) + ($P_{aO_{2}}$ × 0.003), where $C_{aO_{2}}$ is arterial oxygen content (mL O_2_/dL), Hb is haemoglobin concentration (g/dL), 1.34 is the mL O_2_/g Hb, $S_{aO_{2}}$ is arterial oxygen saturation (%), $P_{aO_{2}}$ is the partial pressure of oxygen (mmHg), and 0.003 is the solubility coefficient of oxygen. $P_{aO_{2}}$ was assumed to be 100 mmHg in all participants. $C_{aO_{2}}$ could not be computed in two females due to an inability to collect a blood sample. To compute oxygen delivery, $C_{aO_{2}}$ was multiplied by $\dot{Q}$. $\dot{Q}$ was measured using Doppler echocardiography, as a product of HR and SV. Blood volume was estimated using the Nadler equation (Nadler et al., 1962), which was developed in physiologically normal adults aligning with the present study. In males, blood volume was calculated as: blood volume = (0.3369 × height^3^) + (0.03219 × weight) + 0.6041. In females, blood volume was calculated as: blood volume = (0.3561 × height^3^) + (0.03308 × weight) + 0.1833.

#### 2.8.6 Interpolation of data to a fixed HR of 120 bpm

Linear interpolations were completed to match the period of diastole and ventricular filling across all participants, given that previous work found heterogeneity in $\dot{Q}$–${\dot{V}}_{O_{2}}$ attributable to SV at a HR of 120 bpm (Bentley et al., 2018). For each participant, echocardiographic parameters obtained that were nearest to approaching and exceeding a HR of 120 bpm were interpolated. It is acknowledged that cardiac responses during graded exercise may not be strictly linear (Stohr et al., 2011), and this interpolation approach may introduce an underestimation in a subset of individuals nearing peak effort. Interpolation of echocardiographic values at 120 bpm was feasible in all but three males, as they did not reach a HR greater than 120 bpm during the final stage of echocardiography image acquisition (120 W). GLS at 120 bpm could not be identified in five individuals due to cardiac motion artifacts that resulted in speckle dropout.

### 2.9 Statistical analysis

Statistical analyses were performed using SPSS Statistics software version 28 (IBM Corp., Armonk, NY, USA). All physiological measures were time aligned and analysed to assess the steady state response at rest and during exercise at each intensity. Data were assessed for normality using a Shapiro–Wilk test. Any non‐normally distributed data are presented as median, interquartile range (Q1–Q3) while normally distributed data are presented as the mean ± SD.

A repeated measures mixed model analysis of variance (ANOVA) was used to analyse continuous variables, with the main effect of intensity (rest, 40 W, 80 W or Δ40 W and Δ80 W) and biological sex (male, female). For the ANOVA, homogeneity of variance was assessed by Levene's test. Sphericity was assessed by a Mauchly's sphericity test. If there was a lack of sphericity, a Greenhouse–Geisser correction was applied. Only significant *F*‐statistics within the ANOVA were further assessed using a Bonferroni corrected *post hoc t*‐test during exercise stages. Participant characteristics were assessed between males and females using independent Student's *t*‐test or a Mann–Whitney *U*‐test as appropriate.

Pearson or Spearman correlation analyses were completed, as appropriate, to assess relationships between cardiac structure and function, $\dot{Q}$, SV, ${\dot{V}}_{O_{2}}$, and $\dot{Q}$–${\dot{V}}_{O_{2}}$ with Bonferroni corrections applied for multiple exercise intensities. Interpretation of significant correlations were determined according to Cohen's power primer, where *r* ≤ 0.10 is a small effect size, *r* = 0.30 is a medium effect size, and *r* ≥ 0.50 is a large effect size (Cohen, 1992). Multivariable regressions were performed with a stepwise entry method to determine predictors of the slope of the $\dot{Q}$–${\dot{V}}_{O_{2}}$ relationship, as well as explore predictors of SV heterogeneity at a fixed HR of 120 bpm. Variables were selected a priori to explore biological sex, $C_{aO_{2}}$, EDV (as a surrogate measure of preload), GLS (as a surrogate measure of contractility) and SBP (as a surrogate measure of afterload) to predict SV and $\dot{Q}$–${\dot{V}}_{O_{2}}$ heterogeneity. Assumptions for the regression models were met including linearity, independence of residuals and normal distribution of residuals. The 120 W stage elicited a relative intensity that was ≥90% of ${\dot{V}}_{O_{2}}$ peak in 16 participants and was not included in subsequent analyses. Comparisons with a *P*‐value <0.05 were considered statistically significant.

## 3 RESULTS

### 3.1 Participant characteristics

Participant characteristics are presented in Table 1. Most participants were Caucasian (54%) or Asian (35%), with a minority being other ethnicities (11%; Middle Eastern, African American, Hispanic). Male participants were taller, heavier, and had a greater body surface area, [Hb], $C_{aO_{2}}$ and ${\dot{V}}_{O_{2}}$ peak than females (all *P *< 0.030). [Hb] in females had a range of 12.5–15.6 g/dL, and [Hb] in males had a range of 14.4–18.1 g/dL. Female participants had a greater skinfold thickness at the NIRS placement site on the vastus lateralis (*P *< 0.0001) while there was no difference in weekly METs between males and females (270 ± 24 vs. 262 ± 22 METs/week; *P* = 0.245).

**TABLE 1:** Participant characteristics.

Variable	All (*n* = 42)	Male (*n* = 21)	Female (*n* = 21)
Age (years)	24 (22–29)	23 (21–30)	24 (22–28)
Height (cm)	170 ± 9	177 ± 6	164 ± 4^*^
Weight (kg)	69 ± 13	77 ± 11	62 ± 9^*^
BSA (m^2^)	1.80 ± 0.19	1.94 ± 0.15	1.67 ± 0.13^*^
BMI (kg/m^2^)	23.7 ± 2.8	24.4 ± 2.4	22.9 ± 3.1
ATT (mm)	6 (4–9)	5 (3–6)	9 (8–10)^*^
Hemoglobin (g/dL)	15.1 ± 1.4	16 ± 1.1	14.1 ± 0.9^*^
$C_{aO_{2}}$ (mL O_2_/dL)	20.29 ± 1.79	21.43 ± 1.46	19.03 ± 1.18^*^
METs (METs/week)	266 ± 23	270 ± 24	262 ± 22
${\dot{V}}_{O_{2}}$ peak (mL/kg/min)	35.0 ± 7.9	37.6 ± 9.0	32.4 ± 5.7^*^

*Note*: Normal values are presented as means ± SD. Non‐normal values are presented as median (interquartile range; Q1–Q3)

* Statistically significant difference between males and females, *P *< 0.05. ATT, adipose tissue thickness at the vastus lateralis; BMI, body mass index; BSA, body surface area; $C_{aO_{2}}$ (*n* = 40), arterial oxygen content; METs, metabolic equivalent of task, ${\dot{V}}_{O_{2}}$ peak, peak rate of ${\dot{V}}_{O_{2}}$.

### 3.2 Slope of the $\dot{Q}$–${\dot{V}}_{O_{2}}$ relationship

#### 3.2.1 Heterogeneity in the slope of the $\dot{Q}$–${\dot{V}}_{O_{2}}$ relationship

**FIGURE 2:**
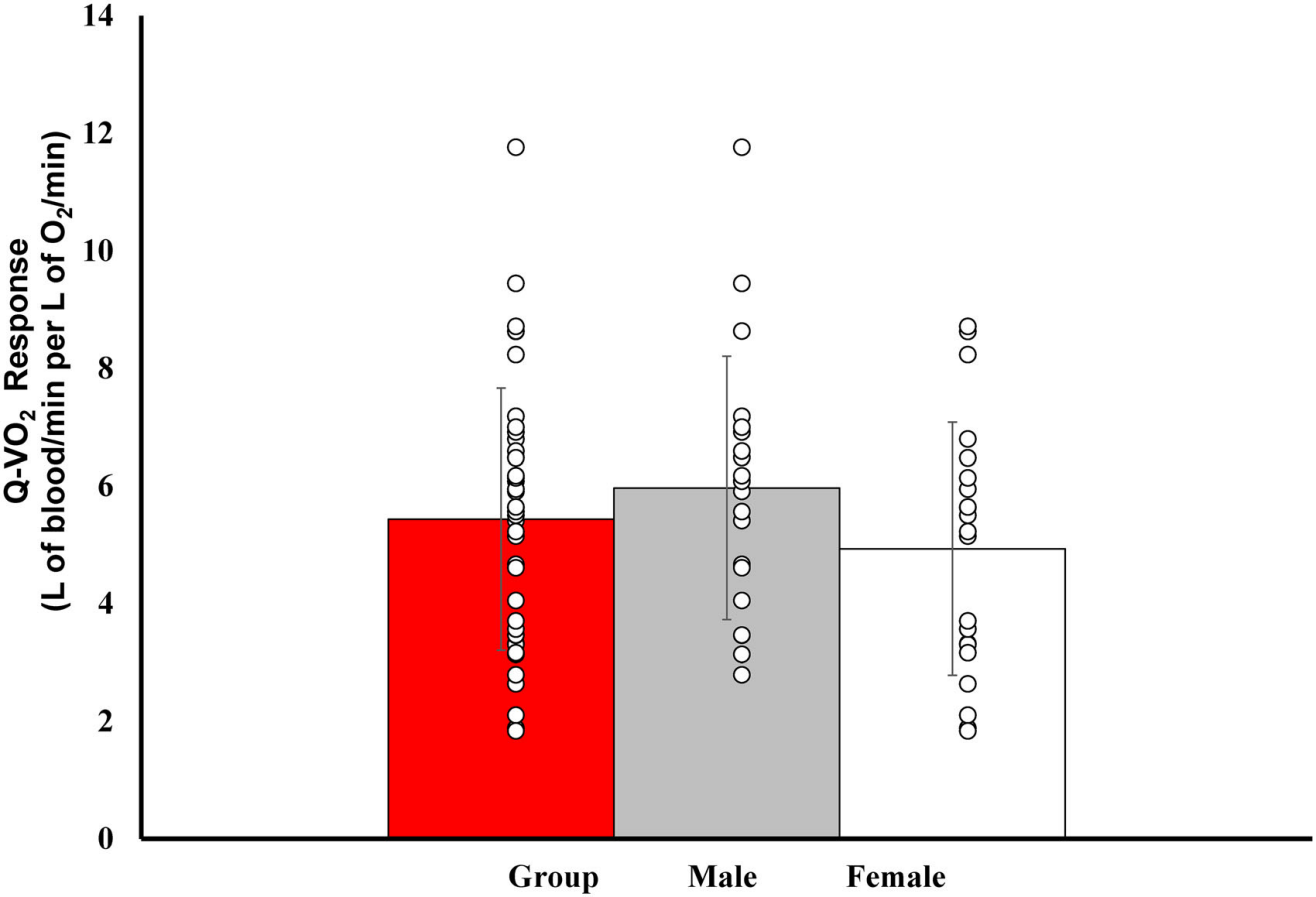
Individual Δ$\dot{Q}$–${\dot{V}}_{O_{2}}$ responses. The increase in cardiac output ($\dot{Q}$) required for a 1 L/min increase in ${\dot{V}}_{O_{2}}$ from 40 to 80 W (*n* = 41). Open circles represent individual responses.

The slope of the $\dot{Q}$–${\dot{V}}_{O_{2}}$ relationship ranged from 1.8 to 11.8 L of blood/min per L of O_2_/min (Figure 2). Males and females did not significantly differ (6.0 ± 2.2 vs. 4.9 ± 2.2 L blood/min per L of O_2_/min; *P* = 0.140). Examining potential predictors of the $\dot{Q}$–${\dot{V}}_{O_{2}}$ relationship, $\dot{Q}$–${\dot{V}}_{O_{2}}$ was correlated with SV at 80 W (*r* = 0.428, *P* = 0.005), but not with HR (*r* = −0.090, *P* = 0.575). In addition, though males had a significantly greater estimated blood volume compared to females (*P *< 0.0001), estimated blood volume was not correlated to $\dot{Q}$–${\dot{V}}_{O_{2}}$ when looked at as a group (*r* = 0.284, *P* = 0.072), or in males (*r* = 0.393, *P* = 0.086) or females (*r* = −0.137, *P* = 0.555). Relative heterogeneity in $\dot{Q}$–${\dot{V}}_{O_{2}}$ was also not different between males and females (110 ± 41% vs. 91 ± 40%) (*P* = 0.140). When $\dot{Q}$–${\dot{V}}_{O_{2}}$ was assessed in a subset of participants up to 120 W (*n* = 25), interpretation did not change.

#### 3.2.2 $C_{aO_{2}}$ and Δ$S_{mO_{2}}$ are correlated to the slope of $\dot{Q}$–${\dot{V}}_{O_{2}}$ relationship in females

**FIGURE 3:**
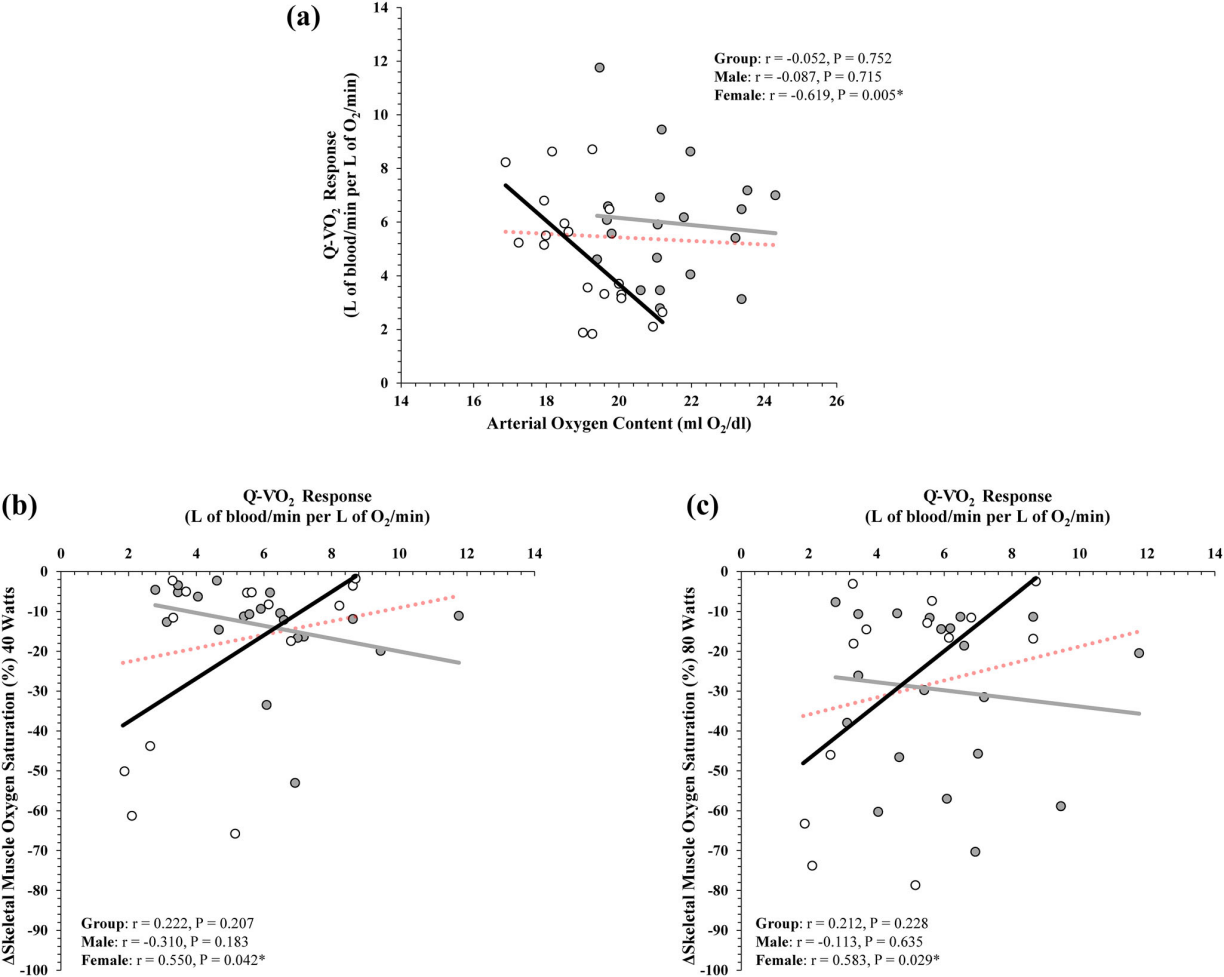
Relationship between $C_{aO_{2}}$ and Δ$S_{mO_{2}}$ to Δ$\dot{Q}$–${\dot{V}}_{O_{2}}$. (a) Correlation between $C_{aO_{2}}$ and Δ$\dot{Q}$–${\dot{V}}_{O_{2}}$ (*n* = 39 group; *n* = 20 male and *n* = 19 female). (b) Correlation between Δ$S_{mO_{2}}$ at 40 W and Δ$\dot{Q}$–${\dot{V}}_{O_{2}}$ (*n* = 34 group; *n* = 20 male and *n* = 14 female). (c) Correlation between Δ$S_{mO_{2}}$ at 80 W and Δ$\dot{Q}$–${\dot{V}}_{O_{2}}$ (*n* = 34 group; *n* = 20 male and *n* = 14 female). Female participants are presented as white circles; male participants are presented as grey circles. Female regression (black), male regression (grey) and group regression (red) are presented as individual lines of best fit.

When analysed as a group, $C_{aO_{2}}$ was not a predictor of the $\dot{Q}$–${\dot{V}}_{O_{2}}$ relationship (*r* = −0.052, *P* = 0.752); however, further inspection revealed that while there was no relationship in males (*r* = −0.087, *P* = 0.715), there was a strong negative correlation between $C_{aO_{2}}$ and the slope of the $\dot{Q}$–${\dot{V}}_{O_{2}}$ relationship (*r* = −0.619, *P* = 0.005) in females (Figure 3a). In addition, when analysed as a group, Δ$S_{mO_{2}}$ at both 40 W and 80 W was not predicted by the $\dot{Q}$–${\dot{V}}_{O_{2}}$ relationship (all *r* < 0.222, all *P* > 0.207) (Figure 3b,c). Analysis by sex revealed no relationship in males (all *r* < −0.310, all *P* > 0.183), though, a strong positive correlation with the slope of the $\dot{Q}$–${\dot{V}}_{O_{2}}$ relationship at 40 W (*r* = 0.550, *P* = 0.042) and at 80 W (*r* = 0.583, *P* = 0.029) in females.

#### 3.2.3 Exploration of indices predicting SV and ΔSV at a fixed HR

A stepwise regression was completed with biological sex, $C_{aO_{2}}$, EDV, GLS and SBP at a fixed HR of 120 bpm to predict the SV heterogeneity. The regression model is presented in Table 2 while the physiological data is presented in Appendix Table A1. The final regression model included EDV, GLS and biological sex (*F* = 36.11; *P *< 0.0001, *r^2^* = 0.800), but not SBP or $C_{aO_{2}}$. A second stepwise regression was also completed with biological sex, $C_{aO_{2}}$, EDV, GLS and SBP at a fixed HR of 120 bpm to predict the ΔSV heterogeneity (Table 2). The final model only included EDV (*F* = 12.34; *P* = 0.001, *r^2^* = 0.298).

**TABLE 2:** Regression models for prediction of SV and ΔSV at a heart rate of 120 bpm.

	B	SE B	β	*P*
Predictors of SV at 120 bpm				
Constant	2.17	15.83		
EDV, 120 bpm	0.43	0.08	0.585	<0.0001
GLS, 120 bpm	−2.64	0.52	−0.478	<0.0001
Sex	−17.12	4.92	−0.414	0.002

Predictors of ΔSV at 120 bpm				
Constant	1.86	4.78		
EDV, 120 bpm	0.18	0.05	0.546	0.001

*Note*: Regression models for predicting SV at a heart rate of 120 bpm (*r^2^* = 0.800; *P *< 0.0001; *n* = 31) and ΔSV at 120 bpm (*R^2^* = 0.298; *P* = 0.001; *n* = 31). bpm, beats per minute; EDV, end‐diastolic volume; GLS, global longitudinal strain.

### 3.3 Physiological responses to semi‐upright cycling exercise

#### 3.3.1 HR, SV and cardiac $\dot{Q}$

Absolute measures of HR, SV and $\dot{Q}$ are presented in Table 3, and measures indexed to BSA are presented in Appendix Table A2. HR increased from rest throughout exercise, with greater HRs in females compared to males (all *P *< 0.0001). Both SV (*P *< 0.0001) and $\dot{Q}$ increased from rest throughout exercise (all *P *< 0.0001) with greater values in males compared to females (both *P *< 0.017). When analysing values as a Δ from rest, sex differences were lost for SV (*P* = 0.121) and $\dot{Q}$ (*P* = 0.452), though, the effect of intensity persisted for ΔSV and Δ$\dot{Q}$ (both *P *< 0.0001).

**TABLE 3:** Physiological absolute and delta (Δ) response to cycling exercise.

Variable	Group (*n* = 42)			Male (*n* = 21)			Female (*n* = 21)		
	Rest	40 W	80 W	Rest	40 W	80 W	Rest	40 W	80 W
Absolute									
HR (bpm)^#^	68 ± 11	102 ± 15	127 ± 20	63 ± 10^a^^,^^b^	92 ± 11^c^	113 ± 16	73 ± 10^*^^,^^a^^,^^b^	112 ± 13^*^^,^^c^	139 ± 15^*^
SV (mL)^+m,^^	62 ± 18^a^^,^^b^	75 ± 19^c^	80 ± 21	73 ± 21	87 ± 19	94 ± 21	53 ± 8	64 ± 9	68 ± 11
$\dot{Q}$ (L/min)^+m,^^	4.1 ± 0.9^a^^,^^b^	7.5 ± 1.3^c^	9.9 ± 1.7	4.5 ± 1.1	7.9 ± 1.5	10.4 ± 1.9	3.8 ± 0.5	7.1 ± 0.9	9.3 ± 1.4
${\dot{V}}_{O_{2}}$ (L/min)^+m,^^	0.29 ± 0.08^a^^,^^b^	0.89 ^,^± 0.17^c^	1.3 ± 0.16	0.32 ± 0.09	0.94 ± 0.16	1.38 ± 0.16	0.26 ± 0.05	0.85 ± 0.17	1.29 ± 0.16
%${\dot{V}}_{O_{2}}$ peak (%)^#^	12.3 ± 2.9	39.1 ± 11.0	58.9 ± 15.6	11.6 ± 3.1 ^a^^,^^b^	34.5 ± 9.8 ^c^	50.8 ± 14.8	13.1 ± 2.5 ^a^^,^^b^	43.6 ± 10.4^*,c^	66.9 ± 12.1^*^
Oxygen delivery (mL O_2_/min)^#^	840 ± 201	1529 ± 283	2000 ± 369	957 ± 200^a^^,^^b^	1690 ± 270^c^	2227 ± 341	718 ± 110^*a^^,^^b^	1361 ± 183^*^^,^^c^	1761 ± 216^*^
a‐vO_2_ difference (mL O_2_/L)^^^	71 ± 18^a,b^	121 ± 25^c^	139 ± 24	75 ± 20	123 ± 26	136 ± 21	69 ± 16	120 ± 24	142 ± 27

Delta (Δ) from rest									
ΔHR (bpm)^#^	—	34 ± 10	58 ± 15	—	29 ± 8^a^	50 ± 13	—	39 ± 9^*^^,^^a^	67 ± 12^*^
ΔSV (mL)^^^	—	12.9 ± 10.2^a^	18.0 ± 13.4	—	14.5 ± 12.4	21.3 ± 10.8	—	11.5 ± 7.5	14.8 ± 9.1
Δ$\dot{Q}$ (L/min)^^^	—	3.4 ± 1.0^a^	5.7 ± 1.3	—	3.5 ± 1.1	6.0 ± 1.3	—	3.4 ± 1.0	5.5 ± 1.4
Δ${\dot{V}}_{O_{2}}$ (L/min)^^^	—	0.6 ± 0.1^a^	1.1 ± 0.1	—	0.6 ± 0.1	1.1 ± 0.1	—	0.6 ± 0.1	1.1 ± 0.1
ΔOxygen delivery (mL O_2_/min)^#^	—	688.86 ± 186.71	1159.75 ± 260.93	—	732.61 ± 208.25^a^	1270.19 ± 245.66	—	642.81 ± 153.13^a^	1043.50 ± 228.51^*^
Δ$S_{mO_{2}}$ (%)^#^	—	−10.7 (−16.5 to −5.3)	−16.9 (−45.9 to −11.5)	—	−11.1 (−14.6 to −5.4)^a^	−20.5 (−45.7 to −11.6)	—	−8.4 (−37.2 to −5.1)	−15.6 (−39.0 to −8.4)
Δa‐vO_2_ difference (mL O_2_/L)^^^	—	50 ± 27^a^	67 ± 26	—	48 ± 31	61 ± 26	—	51 ± 24	73 ± 25
ΔO_2_ cost (O_2_/watt)^^^	—	15.3 ± 3.2^a^	13.2 ± 1.6	—	15.5 ± 3.3	13.2 ± 1.6	—	15.0 ± 3.2	13.2 ± 1.6

*Note*: Values are presented as means ± SD. Delta (Δ) values are a change from rest.

* Significant difference *P *< 0.05 for males and females at a given intensity

^+m^ main effect of sex *P *< 0.05 and male values are greater

^+f^ Main effect of sex *P *< 0.05 and female values are greater

^^^ main effect of intensity *P *< 0.05

^#^ significant interaction at the *P *< 0.05 level. For absolute values

^a^ difference between rest and 40 W

^b^ difference between rest and 80 W

^c^ difference between 40 W and 80 W (all *P *< 0.05). For delta values, a represents a difference between 40 W and 80 W (*P *< 0.05). Group *n* = 42 unless otherwise noted. ΔO_2_ cost, oxygen cost; Δ$S_{mO_{2}}$, skeletal muscle oxygen saturation (*n* = 35); HR, heart rate (*n* = 41); $\dot{Q}$, cardiac output (*n* = 41); SV, stroke volume (*n* = 41); ${\dot{V}}_{O_{2}}$, rate of oxygen consumption (*n* = 39); a‐vO_2_ difference, arterial–venous oxygen difference (*n* = 41).

#### 3.3.2 ${\dot{V}}_{O_{2}}$, oxygen delivery/cost and a‐vo_2_ difference

Measures of whole body ${\dot{V}}_{O_{2}}$ and oxygen delivery are presented in Table 3. ${\dot{V}}_{O_{2}}$ increased from rest with each exercise intensity (all *P *< 0.0001) with males having a greater absolute ${\dot{V}}_{O_{2}}$ (*P* = 0.028). When expressed as a Δ from rest, ${\dot{V}}_{O_{2}}$ increased with exercise intensity (*P *< 0.0001) with no difference between males and females (*P* = 0.770) (Table 3). Average relative ${\dot{V}}_{O_{2}}$ peak was 35.0 ± 7.9 mL/kg/min and was not related to the $\dot{Q}$–${\dot{V}}_{O_{2}}$ relationship on a group level (*r* = −0.014, *P* = 0.930), nor when analysed by sex in males (*r* = −0.309, *P* = 0.185) or females (*r* = 0.225, *P* = 0.327).

Convective oxygen delivery increased with exercise intensity in both sexes (all *P *< 0.0001) while being greater in males at rest, 40 W, and 80 W compared to females (all *P *< 0.0001). These observations persisted when oxygen delivery was expressed as a Δ from rest (all *P *< 0.005) except there was no difference between males and females at 40 W (*P* = 0.135). The Δ in oxygen delivery per watt (mL O_2_/min/W) increased with exercise intensity (*P *< 0.0001) and was greater in males compared to females (*P* = 0.030). O_2_ cost and a‐vO_2_ difference (*P *< 0.0001) increased from rest to exercise, but were not different between males and females (all *P* > 0.704).

#### 3.3.3 Skeletal muscle saturation

Δ$S_{mO_{2}}$ differed between 40 W and 80 W in males (*P *< 0.0001), while females did not differ (*P* = 0.116). Males and females did not differ at 40 W (*P* = 0.212) or at 80 W (*P* = 0.614) (Table 3).

### 3.4 SV constituents

#### 3.4.1 LV volume

EDV (surrogate measure of preload) was greater in males at rest, 40 W and 80 W compared to females (all *P *< 0.0001) with males experiencing an increase in EDV while females did not. ESV was also greater in males (*P *< 0.0001) and decreased from rest to 40 W (*P* = 0.002) and 80 W (*P *< 0.0001) with no difference between 40 W and 80 W (*P* = 0.082). Indexing to BSA did not change interpretations of EDV or ESV (Appendix Table A2). Regarding a change from semi‐upright rest, ΔEDV was greater in males (*P* = 0.006) and increased from 40 W to 80 W (*P* = 0.048), whereas ΔESV decreased (*P* = 0.027).

#### 3.4.2 LV longitudinal strain

LV GLS (surrogate measure of contractility) was analysed in 32 participants. GLS increased from rest (−18.1 ± 3.7%) to 40 W (−22.2 ± 3.9%) and 80 W (−24.1 ± 3.9%) (all *P *< 0.024) with females having greater GLS than males (−23.6 ± 4.8 vs. −19.9 ± 3.8%, *P* = 0.001).

#### 3.4.3 Arterial blood pressure and total vascular conductance

SBP (surrogate measure of afterload) increased from rest (121 ± 17 mmHg) to 40 W (141 ± 18 mmHg) to 80 W (160 ± 20 mmHg) (all *P *< 0.0001) and was higher in males (149 ± 23 vs. 133 ± 23 mmHg, *P* = 0.002). Additionally, MAP followed a similar pattern as SBP (all *P *< 0.018) while ΔMAP increased from 40 W to 80 W (*P* = 0.006). Lastly, total vascular conductance (TVC) increased from rest at 40 W and 80 W (all *P *< 0.0001) as did ΔTVC (*P *< 0.0001), though males and female were not different (*P* = 0.534).

## 4 DISCUSSION

Variability in the slope of the $\dot{Q}$–${\dot{V}}_{O_{2}}$ relationship may arise due to SV heterogeneity (Bentley et al., 2018) and difference in cardiac structure and function between males and females; however, this was previously contentious. The primary novel findings of the present study were: (1) in females, Δ$S_{mO_{2}}$ had a strong positive correlation with the slope of the $\dot{Q}$–${\dot{V}}_{O_{2}}$ relationship and $C_{aO_{2}}$ had a strong negative correlation with the slope of the $\dot{Q}$–${\dot{V}}_{O_{2}}$ relationship; (2) though males and females did not significantly differ in the $\dot{Q}$–${\dot{V}}_{O_{2}}$ slope or relative heterogeneity in the relationship, there was a considerable range in the slope of the $\dot{Q}$–${\dot{V}}_{O_{2}}$ relationship across the group (1.8–11.8 L of blood/min per L of O_2_/min; and (3) SV heterogeneity at a fixed HR of 120 bpm was partially explained by EDV, GLS and biological sex, but not SBP and $C_{aO_{2}}$.

### 4.1 Predicting the slope of $\dot{Q}$–${\dot{V}}_{O_{2}}$ relationship and functional implications

Interindividual variability in the slope of the $\dot{Q}$–${\dot{V}}_{O_{2}}$ relationship may reflect differences in convective oxygen delivery and $C_{aO_{2}}$ (Adami et al., 2014) with previous research in females identifying a strong, negative correlation between $C_{aO_{2}}$ and $\dot{Q}$ that appeared present in the anaemic [Hb] range of 10–13 g/dL; however, upon closer inspection, this relationship was seemingly absent in those with normal (13–17 g/dL) [Hb] (Freedson et al., 1981). While the present findings in females ([Hb]; 12.5–15.6 g/dL) support this relationship, we observed no relationship between $C_{aO_{2}}$ and the slope of the $\dot{Q}$–${\dot{V}}_{O_{2}}$ relationship in males ([Hb]; 14.4–18.1 g/dL). That said, this lack of relationship in males fully aligns with prior observations in males (Bentley et al., 2018) and may suggest that the ability to match oxygen delivery to skeletal muscle oxygen demand may be more contingent upon $\dot{Q}$ in females. In light of [Hb] differences between males and females, it is noteworthy that females presented with an attenuated increase in oxygen delivery up to 450 mL O_2_/min at a submaximal intensity of 80 W, suggesting that $\dot{Q}$ was not elevated to maintain the rate of convective oxygen delivery. In support of previous work (Bentley et al., 2018), and in line with our hypotheses, we observed no relationship between Δ$S_{mO_{2}}$ and the slope of the $\dot{Q}$–${\dot{V}}_{O_{2}}$ relationship in males. However, contrary to our hypotheses, saturation at the vastus lateralis was significantly lower in those with a lower $\dot{Q}$–${\dot{V}}_{O_{2}}$ relationship in females. Interestingly, despite no sex difference in the $\dot{Q}$–${\dot{V}}_{O_{2}}$ slope, it may be inferred then that males and females appear to differ in vastus lateralis muscle blood flow (MBF), although MBF was not assessed in the present study. Previous work in males has shown greater computed $\dot{Q}$ redistribution in individuals with a lower $\dot{Q}$–${\dot{V}}_{O_{2}}$ slope (Bentley et al., 2018). A possible explanation in males may be that the apparent mechanism for the close control of oxygen delivery and Δ$S_{mO_{2}}$ resides in the ability to recruit MBF through $\dot{Q}$ redistribution, rather than augment $\dot{Q}$, though this would need to be examined further. Interestingly, the functionality of this mechanism may be different in females, though this remains to be confirmed.

Collectively, these findings may indicate that males and females achieve similar $\dot{Q}$–${\dot{V}}_{O_{2}}$ slopes through different physiological strategies. Specifically, in males, we hypothesize that the ability to redistribute $\dot{Q}$ toward active skeletal muscle to maintain oxygen delivery to demand matching may help preserve Δ$S_{mO_{2}}$ during submaximal intensities, even when overall oxygen delivery is challenged. However, in females, the relationships between $C_{aO_{2}}$, Δ$S_{mO_{2}}$ and the $\dot{Q}$–${\dot{V}}_{O_{2}}$ slope suggests that $\dot{Q}$ plays a more important role in controlling oxygen delivery compared to males, particularly in females with lower [Hb]. Indeed, in females these findings also suggest that increases in $\dot{Q}$ are not always sufficient such that local skeletal muscle Δ$S_{mO_{2}}$ may not be maintained given the strong, negative relationship between the $\dot{Q}$–${\dot{V}}_{O_{2}}$ slope and vastus lateralis Δ$S_{mO_{2}}$.

### 4.2 Range of interindividual differences in $\dot{Q}$–${\dot{V}}_{O_{2}}$

Previous work examining the slope of the $\dot{Q}$–${\dot{V}}_{O_{2}}$ relationship using acetylene rebreathing or CO_2_ rebreathing techniques reported $\dot{Q}$ increases of ∼5–7 L/min per litre increase in ${\dot{V}}_{O_{2}}$ (Adami et al., 2014; Faulkner et al., 1977; Proctor et al., 1998), while conflicting reports using inert‐gas rebreathing and dilution techniques found variation in $\dot{Q}$ up to ∼5 L/min at any given rate of ${\dot{V}}_{O_{2}}$ (Astrand et al., 1964; Bentley et al., 2018, 2019; Reeves et al., 1961; Yamaguchi et al., 1986). In the present study, the slope of the $\dot{Q}$–${\dot{V}}_{O_{2}}$ relationship for the whole group had a range of 1.8–11.8 L of blood/min per L of O_2_/min and contrary to our hypothesis, variability was not different in males and females (2.8–11.8 L of blood/min per L of O_2_/min vs. 1.8–8.7 L of blood/min per L of O_2_/min; *P* = 0.732). In order to identify a significant difference with a power of 0.8 and α of 0.05, 128 males and females would be required. The present absence of a difference between males and females aligns with previous work in chronically endurance trained individuals (Proctor et al., 1998). Notably, we observed that ${\dot{V}}_{O_{2}}$ peak was not correlated with METs from the 7‐day physical activity recall questionnaire or with the slope of the $\dot{Q}$–${\dot{V}}_{O_{2}}$ relationship. This suggests that the observed variability is not a product of differences in aerobic fitness. While previous work reported a positive relationship between aerobic fitness and the $\dot{Q}$–${\dot{V}}_{O_{2}}$ slope (Bentley et al., 2018), the semi‐upright cycling posture presently employed is noteworthy since this body position alters central haemodynamics (Hinghofer‐Szalkay, 2011), attenuates lower limb perfusion pressure and has been shown to elicit a reduced ${\dot{V}}_{O_{2}}$ at a given workload compared to traditional upright cycling (Bentley et al., 2025).

The range found in the slope of the $\dot{Q}$–${\dot{V}}_{O_{2}}$ relationship from our study extends beyond both the upper and lower bounds of previous findings (Adami et al., 2014; Astrand et al., 1964; Bentley et al., 2018, 2019; Faulkner et al., 1977; Proctor et al., 1998; Reeves et al., 1961; Yamaguchi et al., 1986), and this did not change whether the slope was derived from the entire cohort between 40 and 80 W, or from a limited sample (*n* = 25) between 40 and 120 W because the relative intensity at 120 W was ≥90% of ${\dot{V}}_{O_{2}}$ peak in 16 participants. Importantly, the $\dot{Q}$–${\dot{V}}_{O_{2}}$ slope reflects the increase in $\dot{Q}$ required for a 1 L/min increase in ${\dot{V}}_{O_{2}}$. Since the slope is scaled to a ${\dot{V}}_{O_{2}}$ of 1 L/min rather than absolute exercise intensity, additional scaling is not warranted. To our knowledge, this was the first study to assess $\dot{Q}$ and the slope of the $\dot{Q}$–${\dot{V}}_{O_{2}}$ relationship using echocardiography and the Doppler method. Doppler echocardiography underestimates $\dot{Q}$ at supine rest, and during both seated submaximal (Espersen et al., 1995) and graded maximal (Christie et al., 1987) exercise compared to thermodilution (Christie et al., 1987; Espersen et al., 1995) and inert gas rebreathing (Espersen et al., 1995) – both of which were used in previous studies exploring the slope of the $\dot{Q}$–${\dot{V}}_{O_{2}}$ relationship (Adami et al., 2014; Bentley et al., 2018, 2019; Proctor et al., 1998). As a result of these differing methodologies, $\dot{Q}$–${\dot{V}}_{O_{2}}$ slopes may be methodologically reduced in our study, thus extending below previous ranges, and this would apply to all participants. Despite this measurement‐method‐induced reduction, the measurement of $\dot{Q}$ in the present study had a CV of 5.1% across all measurement periods (HR 68–151 bpm). This variation aligns with previous work applying the Doppler technique during cycling exercise (CV of 5.7%) (Espersen et al., 1995) and thermodilution (6%) (Espersen et al., 1995), though it is higher than the inert gas rebreathing method (2.2%) (Bentley et al., 2018). As such, the presently observed results may be interpreted with a similar level of confidence as previous work.

In addition, exercise was performed in the semi‐upright position in the present study to facilitate the completion of concomitant echocardiography. This position elicits unique haemodynamic conditions compared to that of traditional upright cycling, which was applied in previous investigations (Adami et al., 2014; Astrand et al., 1964; Bentley et al., 2018, 2019; Faulkner et al., 1977; Proctor et al., 1998; Yamaguchi et al., 1986), due to postural‐induced gravitational effects on both central and peripheral haemodynamics (Hinghofer‐Szalkay, 2011). Previous work from our lab found that exercising in a semi‐upright body position results in an increased $\dot{Q}$, yet a decreased ${\dot{V}}_{O_{2}}$ at the same submaximal exercise intensity compared to traditional upright cycling (Bentley et al., 2025). This increase in $\dot{Q}$ was due to elevations in SV as the modulating effect of gravity reducing venous return and impairing LV filling is mitigated when semi‐upright. Our lab hypothesized that the reduction in ${\dot{V}}_{O_{2}}$ in the semi‐upright body position was attributed to postural muscle unloading given that the back is fully supported while semi‐upright, but spinal accessory muscles may be activated while upright. As a result of disparate effects elevating $\dot{Q}$ and reducing ${\dot{V}}_{O_{2}}$ while semi‐upright, slopes of the $\dot{Q}$–${\dot{V}}_{O_{2}}$ relationship will be increased and may explain the elevated range observed in the present study compared to previous work. Providing further support to the present results, the calculated O_2_ cost during exercise at each stage aligns with values reported in previous investigations involving semi‐upright cycling (Bentley et al., 2025). Further, our calculated a‐vO_2_ differences align within previously reported physiological ranges for recreationally active individuals (Mortensen et al., 2005). We observed an a‐vO_2_ difference of ∼121 mL O_2_/L at 39% of ${\dot{V}}_{O_{2}}$ peak, ∼139.0 mL O_2_/L at 59% of ${\dot{V}}_{O_{2}}$ peak, and ∼146 mL O_2_/L at 78% of ${\dot{V}}_{O_{2}}$ peak. This closely aligns with Mortensen et al. (2005) who observed an a‐vO_2_ difference of ∼125 mL O_2_/L at 40% of cycling peak power, ∼140 mL O_2_/L at 60% of cycling peak power, and ∼150 mL O_2_/L 80% of cycling peak power. At the highest intensity of 120 W, though not analysed and presented as noted, we observed an a‐vO_2_ difference range spanning 90–220 mL O_2_/L. In this case, the individual with an a‐vO_2_ difference of 220 mL O_2_/L was at exhaustion, while the individual with an a‐vO_2_ difference 90 mL O_2_/L was still submaximal. The computed upper 95% confidence limit for a‐vO_2_ difference from Mortensen et al. (2005) at exhaustion was 222 mL O_2_/L, which aligns with our present observations. Combined, these outcomes corroborate our measured $\dot{Q}$–${\dot{V}}_{O_{2}}$ slopes and suggest that the observed variability in this relationship stems from inherent, between‐participant differences in mechanisms contributing to oxygen delivery: demand matching, rather than physiological measurement error.

### 4.3 Constituents influencing SV and the $\dot{Q}$–${\dot{V}}_{O_{2}}$ response during exercise

In the present study, SV at 80 W was positively correlated with the slope of the $\dot{Q}$–${\dot{V}}_{O_{2}}$ relationship while HR was not. This coincides with previous observations by Bentley et al. (2018) in recreationally active males. In order to further explore SV heterogeneity in the present study, a fixed HR of 120 bpm was chosen for analysis given the sensitivity of SV to diastolic filling time (Boudoulas et al., 1979). This HR generally aligns with the onset of disparate SV responses between healthy males from Bentley et al. (2018), while also allowing for detailed assessments of diastology prior to the fusion of early and late mitral inflow velocities (Chung et al., 2004).

In the present multivariable regression, we found that EDV, a surrogate for preload, had the greatest significant contribution to SV when accounting for diastolic filling time, confirming our initial hypothesis. Independently, this aligns with previous work demonstrating that preload is primarily responsible for the 30–40% increase in SV at the beginning of whole‐body exercise (Rowland et al., 2003), and supports the increase seen during exercise (Higginbotham et al., 1986). In addition to the importance of preload, contractility (GLS as a surrogate) had the second greatest significant contribution to SV. While elevations in preload may yield increases in contractility and by extension SV, up to the physiological limits of the LV (Kosta & Dauby, 2021), through the Frank–Starling mechanism, contractility may also be independently augmented. At higher exercising HRs when preload and periods of diastole are reduced, the maintenance of SV has been shown to arise from increased preload‐independent contractility of the myocardium, demonstrated by decreases in ESV (Higginbotham et al., 1986). In the present study, contractility was found to increase throughout exercise in both males and females, yet only males were found to also increase preload. This would suggest that males have a greater reliance on the Frank–Starling mechanism to increase SV and $\dot{Q}$ compared to females, which has been previously demonstrated (Wheatley et al., 2014). As more oxygen and energy are required to perform work with increasing exercise intensities, the lack of increase in preload in females in the present study may suggest alternative LV mechanisms responsible for maintaining active muscle oxygen delivery; specifically, a greater reliance on LV contractile function. Indeed, we observed greater contractility in females compared to males at the same absolute exercise intensity, which aligns with previous observations (Lawton et al., 2011). Sex was, however, the smallest contributor to our model though the importance of contractility in the present model aligns with mechanisms of SV augmentation seen with increasing exercise intensities.

While preload and contractility were identified as significant predictors of SV, afterload (SBP as a surrogate) was not and may not have disparately influenced SV in the present study. In isolation, reductions in afterload increase ventricular emptying (Gledhill, 1994), while elevations have the opposite effect such that afterload is inversely related to LV systolic function (MacGregor et al., 1974). In the present study, SBP increased from rest and with each exercise intensity, though the range in elevations varied considerably. ΔSBP had a range of 60 mmHg at 40 W, and 80 mmHg at 80 W. The augmentation of preload and contractility during exercise and their significant contribution in our model could suggest that individuals in the present study were able to overcome the detrimental effect of increases in afterload on SV, at least at intensities up to 120 bpm. While sex was the smallest contributor to our model, males had greater afterload (SBP as a surrogate) at rest and during exercise. This aligns with previous work in healthy, young adult males and females (Alhawari et al., 2018). Collectively, the findings of the present study may suggest that at submaximal intensities, increases in surrogates of preload and contractility are more important determinants of SV heterogeneity than the resistance the LV must overcome to eject blood, though additional work employing direct measures of preload, afterload and contractility may be further explored.

### 4.4 Experimental considerations

As mentioned, our lab previously found that exercising in a semi‐upright body position resulted in an increased $\dot{Q}$ yet a decreased ${\dot{V}}_{O_{2}}$ at the same submaximal exercise intensity (Bentley et al., 2025). This exercise posture may be responsible for the wider range of $\dot{Q}$–${\dot{V}}_{O_{2}}$ relationships in this study compared to previous work (Adami et al., 2014; Astrand et al., 1964; Bentley et al., 2018, 2019; Faulkner et al., 1977; Proctor et al., 1998; Reeves et al., 1961; Yamaguchi et al., 1986). Additionally, although self‐selected pedalling cadence differed between participants due to the allowed range of 65–80 rpm, previous research has found that there is no significant difference in ${\dot{V}}_{O_{2}}$ peak across cycling cadences ranging between 60 and 90 rpm (Knox‐Brown et al., 2025). Participants, as a whole, in the current study presented with a comparatively lower ${\dot{V}}_{O_{2}}$ peak, which may be attributed, in part, to the semi‐upright body position of exercise in comparison to traditional upright cycling (Dillon et al., 2021) and the duration of exercise exceeding the recommended 8–12 min (Buchfuhrer et al., 1983) for ${\dot{V}}_{O_{2}}$ peak identification, though this duration was by design to facilitate submaximal echocardiographic assessment. Further, given the disparate relationship between Δ$S_{mO_{2}}$ and the $\dot{Q}$–${\dot{V}}_{O_{2}}$ slope, an additional consideration may be adipose tissue at the site of NIRS placement. While Δ$S_{mO_{2}}$ measurements from seven females were excluded due to elevated vastus lateralis skinfold thickness (> 12.5 mm), there was no relationship between vastus lateralis skinfold thickness and Δ$S_{mO_{2}}$ (all *P* > 0.505). That said, Δ$S_{mO_{2}}$ may be sensitive to skeletal muscle characteristics and while previous work in males found that fibre type, capillary ratio and enzymatic activity did not relate to Δ$S_{mO_{2}}$ in males (Bentley et al., 2018), the present observations in females warrant further exploration. Menstrual cycle was not controlled for and female participants completed the study across a range of menstrual cycle phases (follicular *n* = 7, luteal *n* = 11, abnormal cycle *n* = 3; 33% oral contraceptive). This suggests that the present findings are more likely attributable to biological sex‐related differences rather than hormonal fluctuations per se.

Hydration status may influence the cardiovascular and metabolic response to exercise; however, participants consumed water ad libitum prior to testing, thus mitigating potential interindividual differences in baseline hydration status. Further, exercise was conducted in a temperature‐controlled laboratory environment in which excessive sweating was limited and while body water loss >2% of body mass has been associated with reductions in SV, elevations in HR and impaired endurance performance (Sawka et al., 2015), this may not be of considerable concern in the present study. That said, due to acquisition limitations, haemoglobin concentration (which is sensitive to plasma volume) was used as a marker of oxygen carrying capacity rather than haemoglobin mass and blood volume was estimated through validated equations. Lastly, the present study performed multivariable regression with five variables included in the initial regression model. General guidelines recommend that at least 10 participants are to be included per independent variable (Green, 1991). With a total sample size of 31 participants, and final models predicting SV and ΔSV comprising three and one independent variable, respectively, the ratio falls within the acceptable range.

### 4.5 Conclusion

During submaximal cycling, we observed a wide range of interindividual differences in the slope of the $\dot{Q}$–${\dot{V}}_{O_{2}}$ relationship, though males and females did not significantly differ. Coinciding with previous work, no relationship was found between $C_{aO_{2}}$ or Δ$S_{mO_{2}}$ and the $\dot{Q}$–${\dot{V}}_{O_{2}}$ slope in males, but a novel negative relationship between $C_{aO_{2}}$, Δ$S_{mO_{2}}$ and the $\dot{Q}$–${\dot{V}}_{O_{2}}$ slope was identified in females. This suggests a close coupling between oxygen delivery and oxygen demand in males, which appears too dependent upon the ability to recruit MBF through $\dot{Q}$ redistribution. However, females appear to match oxygen delivery to oxygen demand through increased peripheral extraction. Additionally, when controlling for HR, SV heterogeneity was explained by EDV, GLS and biological sex, but not SBP or $C_{aO_{2}}$. Future investigations are needed to assess active MBF alongside skeletal muscle fibre characteristics in females to advance the understanding of how oxygen delivery is effectively matched to oxygen demand during physical exertion.

## AUTHOR CONTRIBUTIONS

Adam N. Di Salvo and Robert F. Bentley contributed to the conception or design of the work, acquisition, analysis, or interpretation of data for the work and drafting of the work or revising it critically for important intellectual content. Sinan Osman, Jacob L. Schwartz and Nino Nikolovski contributed to the acquisition, analysis, or interpretation of data for the work and drafting of the work or revising it critically for important intellectual content. All authors approved the final version of the manuscript and agree to be accountable for all aspects of the work in ensuring that questions related to the accuracy or integrity of any part of the work are appropriately investigated and resolved. All persons designated as authors qualify for authorship, and all those who qualify for authorship are listed.

## CONFLICT OF INTEREST

The authors have declared that no competing interests exist. The results of the study are presented clearly, honestly, and without fabrication, falsification, or inappropriate data manipulation.

## GENERATIVE AI STATEMENT

No generative AI tools were used in the preparation of this manuscript.

## Data Availability

The data that support the findings of this study are available from the corresponding author upon reasonable request.

## 1

**TABLE A1:** Functional participant characteristics at a heart rate of 120 bpm.

Variable	Group (*n* = 42)	Male (*n* = 21)	Female (*n* = 21)
SV (mL)	77 ± 20	92 ± 20	65 ± 10*
$\dot{Q}$ (L/min)	8.7 (8.0–10.5)	10.7 (10.0–11.8)	8.7 (8.0–10.5)*
${\dot{V}}_{O_{2}}$ (L/min)	1.28 ± 0.37	1.53 ± 0.35	1.04 ± 0.19*
Oxygen delivery (mL O_2_/min)	1936.15 ± 539.67	2367.02 ± 441.50	1527.97 ± 192.02*
GLS (%)	−24.4 ± 4.2	−21.7 ± 3.7	−25.7 ± 3.8*
EDV (mL)	91.5 ± 30.1	113 ± 30	73 ± 12*

*Note*: Normal values are presented as means ± SD. Non‐normal values are presented as median, interquartile range (Q1–Q3).

* Significant difference *P *< 0.05 for males and females. EDV, end diastolic volume (*n* = 39); GLS, global longitudinal strain (*n* = 34); $\dot{Q}$, cardiac output (*n* = 39); SV, stroke volume (*n* = 39); ${\dot{V}}_{O_{2}}$, rate of oxygen consumption (*n* = 37).

**TABLE A2:** Physiological parameters and left ventricular dimensions and volumes to body surface area.

	Group (*n* = 42)			Male (*n* = 21)			Female (*n* = 21)		
Variable	Rest	40 W	80 W	Rest	40 W	80 W	Rest	40 W	80 W
HR (bpm)^#^	68 ± 11	102 ± 15	127 ± 20	63 ± 10^a^^,^^b^	92 ± 11^c^	113 ± 16	73 ± 10^*^^,^^a^^,^^b^	112 ± 13^*^^,^^c^	139 ± 15^*^
SVi (mL/m^2^)^+m,^^	34 ± 8^a^^,^^b^	42 ± 8^c^	44 ± 9	37 ± 9	45 ± 8	45 ± 8	32 ± 5	39 ± 6	41 ± 6
$\dot{Q}$ i (L/min/m^2^)^^^	2.3 ± 0.4^a^^,^^b^	4.2 ± 0.6^c^	5.5 ± 0.9	2.2 ± 0.4	4.1 ± 0.7	5.4 ± 0.8	2.3 ± 0.3	4.3 ± 0.6	5.6 ± 0.9
IVSdi (cm/m^2^)	0.5 ± 0.1	0.5 ± 0.1	0.5 ± 0.1	0.5 ± 0.1	0.5 ± 0.1	0.5 ± 0.1	0.5 ± 0.1	0.5 ± 0.1	0.5 ± 0.1
LVIDdi (cm/m^2^)^^^	2.4 ± 0.2	2.5 ± 0.3	2.5 ± 0.3	2.4 ± 0.2	2.5 ± 0.2	2.5 ± 0.3	2.4 ± 0.2	2.6 ± 0.2	2.6 ± 0.2
LVPWdi (cm/m^2^)	0.5 ± 0.1	0.4 ± 0.1	0.4 ± 0.1	0.5 ± 0.1	0.4 ± 0.1	0.4 ± 0.1	0.5 ± 0.1	0.4 ± 0.1	0.4 ± 0.1
LVIDsi (cm/m^2^)^^^	1.9 ± 0.2^b^	1.8 ± 0.2	1.7 ± 0.2	1.8 ± 0.2	1.8 ± 0.2	1.7 ± 0.2	1.9 ± 0.2	1.8 ± 0.2	1.8 ± 0.3
LVPWsi (cm/m^2^)	0.5 ± 0.1	0.5 ± 0.1	0.5 ± 0.1	0.5 ± 0.1	0.5 ± 0.1	0.5 ± 0.1	0.6 ± 0.1	0.5 ± 0.1	0.5 ± 0.1
LADi (cm/m^2^)^^^	1.5 ± 0.2^a^^,^^b^	1.5 ± 0.3	1.5 ± 0.3	1.3 ± 0.2	1.5 ± 0.3	1.5 ± 0.3	1.3 ± 0.3	1.5 ± 0.2	1.5 ± 0.2
LVOT Diam i (cm/m^2^)	1.1 ± 0.1	1.1 ± 0.1	1.1 ± 0.1	1.1 ± 0.1	1.1 ± 0.1	1.1 ± 0.1	1.1 ± 0.1	1.1 ± 0.1	1.1 ± 0.1
LVM (g/m^2^)^+m^	72 ± 16			78 ± 16			64 ± 13		
EDVi (mL/m^2^)^#^	48 ± 10	50 ± 11	52 ± 12	52 ± 10^a^^,^^b^	56 ± 12	58 ± 12	43 ± 7^*^	43 ± 5^*^	44 ± 5^*^
ESVi (mL/m^2^)^+m,^^	19 ± 6^a^^,^^b^	17 ± 6	16 ± 6	21 ± 6	19 ± 6	18 ± 6	16 ± 4	14 ± 4	13 ± 4

*Note*: Values are presented as means ± SD.

* Significant difference *P *< 0.05 for males and females at a given intensity

^+m^ main effect of sex *P *< 0.05 and male values are greater

^+f^ main effect of sex *P *< 0.05 and female values are greater

^^^ main effect of intensity *P *< 0.05; #significant interaction at the *P *< 0.05 level

^a^ Difference between rest and 40 W

^b^ difference between rest and 80 W

^c^ difference between 40 W and 80 W (all *P *< 0.05). EDV, end‐diastolic volume (*n* = 40); ESV, end‐systolic volume (*n* = 37); HR, heart rate (*n* = 41); IVSd, interventricular septal wall thickness in diastole (*n* = 33); LAD, left atrial anterioposterior dimension (*n* = 38); LVIDd, LV internal dimension in diastole (*n* = 37); LVIDs, LV internal dimension in systole (*n* = 27); LVM, left ventricular mass (*n* = 39); LVOT Diam, LV outflow tract diameter (*n* = 42); LVPWd, LV posterior wall thickness in diastole (*n* = 37); LVPWs, LV posterior wall thickness in systole (*n* = 26); $\dot{Q}$, cardiac output (*n* = 41); SV, stroke volume (*n* = 41).
